# Should we adjudicate outcomes in stroke trials? A systematic review

**DOI:** 10.1177/17474930221094682

**Published:** 2022-05-10

**Authors:** Peter J Godolphin, Philip M Bath, Alan A Montgomery

**Affiliations:** 1 MRC Clinical Trials Unit at UCL, Institute of Clinical Trials and Methodology, University College London, London, UK; 2 Stroke Trials Unit, Mental Health & Clinical Neuroscience, University of Nottingham, Nottingham, UK; 3 Stroke, Nottingham University Hospitals NHS Trust, Nottingham, UK; 4 Nottingham Clinical Trials Unit, University of Nottingham, Nottingham, UK

**Keywords:** Adjudication, stroke, clinical trial

## Abstract

**Background:**

Central adjudication of outcomes is common in randomized clinical trials in stroke. The rationale for adjudication is clear; centrally adjudicated outcomes should have less random and systematic errors than outcomes assessed locally by site investigators. However, adjudication brings added complexities to a clinical trial and can be costly.

**Aim:**

To assess the evidence for outcome adjudication in stroke trials.

**Summary of review:**

We identified 12 studies evaluating central adjudication in stroke trials. The majority of these were secondary analyses of trials, and the results of all of these would have remained unchanged had central adjudication not taken place, even for trials without sufficient blinding. The largest differences between site-assessed and adjudicator-assessed outcomes were between the most subjective outcomes, such as causality of serious adverse events. We found that the cost of adjudication could be upward of £100,000 for medium to large prevention trials. These findings suggest that the cost of central adjudication may outweigh the advantages it brings in many cases. However, through simulation, we found that only a small amount of bias is required in site investigators’ outcome assessments before adjudication becomes important.

**Conclusion:**

Central adjudication may not be necessary in stroke trials with blinded outcome assessment. However, for open-label studies, central adjudication may be more important.

## Introduction

Randomized trials are commonly placed at the top of the evidence hierarchy.^1^ It is unusual for clinical guidelines to recommend new treatments without at least one randomized trial providing evidence of treatment efficacy. However, it is common for many processes in randomized trials to be undertaken without a strong evidence base to support them. One such process is central adjudication of outcomes.

Central adjudication is common in randomized trials investigating interventions to prevent first or subsequent stroke or to treat patients with acute stroke.^2^ In these trials, outcomes are typically first assessed by local or site investigators at each participating trial site. All or part of the data used in this assessment (e.g. patient phenotypes, brain scans, and video footage) is sent to a central team of experts, referred to as the central adjudicators or blinded endpoint review committee, who provide an additional assessment of the trial outcome. In most cases, the adjudicated assessment of the outcome is used in all subsequent trial analyses, but occasionally outcomes from site investigators and adjudicators are combined to provide a final, joint, outcome.^3^ Typical adjudicated outcomes in stroke trials include stroke,^4^ composite outcomes that include stroke (such as major adverse cardiovascular events),^5,6^ degree of disability (e.g. the modified Rankin Scale, mRS)^7^, and safety outcomes such as adverse event disease classification or likely causality of adverse events.^8^

Adjudication is a contentious topic, with many considering it vital to preserve clinical trial quality,^9,10^ while others deem it an unnecessary additional burden for relatively little benefit.^11^ However, the rationale for central adjudication appears clear. Central adjudicators should always be blinded to treatment assignment, and therefore, adjudication should protect against detection bias and reduce the amount of systematic error (differential misclassification) in the trial outcome.^11^ In addition, adjudication provides a standardized way of assessing outcomes. Having a central team of experts assessing an outcome in a consistent manner should reduce the amount of random error (non-differential misclassification) in the trial outcome when compared with outcomes determined by many local site investigators. Hence, central adjudication should reduce bias and improve statistical power.

Since central adjudication should reduce both differential and non-differential misclassification, we might expect trial results to differ depending on whether the primary outcome is adjudicated. A Cochrane systematic review identified 47 trials across all clinical areas with subjective binary outcomes that were assessed by both central adjudicators and site investigators.^12^ The reviewers re-analyzed each trial using the site-assessed outcome and compared this treatment effect (odds ratio) with the treatment effect obtained using the adjudicated outcome. The resultant ratio of odds ratios (ROR) was 1.00 (95% confidence interval (CI) = 0.97–1.04), indicating no evidence of a difference in treatment effects regardless of the outcome assessment. A further meta-analysis of 10 cardiovascular trials that had adjudicated their primary outcome had similar findings (ROR = 1.00, 95% CI = 0.97–1.02).^13^

In recent years, there has been a plethora of methodological research investigating the benefit of adjudication in stroke trials, including findings from the present authors.^14^ In this systematic review, we aim to identify all the evidence relating to central adjudication in stroke trials and evaluate it collectively to understand the importance of adjudication in different contexts. Based on the evidence presented, we give our suggestions on when to implement adjudication in future trials.

## Methods

This systematic review is reported according to the Preferred Reporting Items for Systematic Reviews and Meta-Analyses (PRISMA) statement.^15^

### Eligibility criteria

To be eligible for this systematic review, studies had to evaluate at least one aspect of central adjudication in randomized trials solely investigating stroke. We refer to these as methodological articles hereafter, as they evaluate trial conduct, rather than patient outcomes. There was no restriction on the year of publication, but articles not in English were recorded but excluded. Methodological articles comparing adjudication to registry information or routine data were also excluded.

### Search strategy and selection criteria

To identify methodological articles, we updated searches used in a previous systematic review^2^ that aimed to identify all records that involved adjudication in stroke trials, with methodological articles a subset of this (see the Supplemental Material for a description of the search terms). The previous systematic review searched from database inception until 6 November 2018. To supplement the previous search, in this current review, we searched PubMed, Embase, and Google Scholar (searches from 6 November 2018 until 12 October 2021; only the first 100 records from Google Scholar screened). Titles and abstracts were screened initially, with full texts only sought for potentially eligible records. Following this, all full texts were assessed for eligibility.

### Data synthesis

In this systematic review, we do not undertake any quantitative synthesis of the data. Instead, we present a narrative summary of the evidence from each of the included methodological studies.

## Results

### Search results

**Figure 1.**
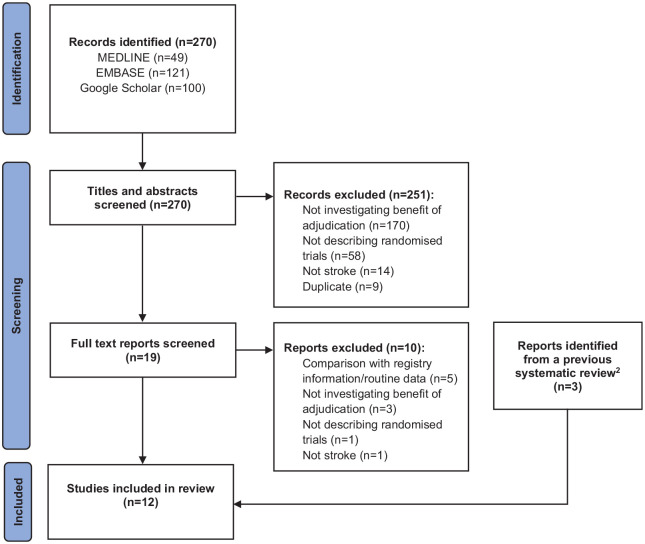
PRISMA flow diagram.

The previous systematic review included three eligible methodological articles, and our new searches identified a further nine studies, resulting in twelve^2,3,14,16–24^ that evaluated the benefit of adjudication in stroke trials (Figure 1). A summary of the eligible methodological studies is given in Table 1.

**Table 1.** Summary of included studies.

Study	Study type	Outcome adjudicated	Summary
Ninomiya et al.^16^	Secondary analysis	Stroke (primary outcome) Stroke type (baseline covariate) Cause of death (safety outcome)	Compared outcomes determined by both adjudicators and site investigators to assess whether adjudication had any impact on the results of a single trial.
McArthur et al.^17^	Simulation study and trial	Functional outcome after stroke (primary outcome)	Simulated scenarios to explore whether increasing mRS reliability translated to increased power. Virtual trial developed that compared mRS scores reported by adjudicators and local site investigators.
López-Cancio et al.^18^	Secondary analysis	Functional outcome after stroke (primary outcome)	Compared adjudicated and site investigator reported mRS scores in a single trial to determine agreement.
Godolphin et al.^3^	Secondary analysis and simulation	Stroke type (baseline covariate)	Investigated whether using site investigator reported stroke type gave different results to adjudicated stroke type in a single trial. Increasing error was simulated in the investigator reported outcome to explore scenarios where site investigators were worse at determining stroke type.
Godolphin et al.^19^	Secondary analysis	Serious adverse events (safety outcome)	Compared serious adverse events determined by site investigators and adjudicators in a single trial.
Easton et al.^20^	Secondary analysis	Composite including stroke (primary outcome) Cause of death (safety outcome) Major bleeding (safety outcome)	Compared outcomes determined by both adjudicators and site investigators to determine whether adjudication had any impact on the results of a single trial.
Godolphin et al.^2^	Systematic review and meta-analysis	Stoke (primary outcome) Composite including stroke (primary outcome) Functional outcome after stroke (primary outcome)	Systematic review of 15 trials. Treatment effect using adjudicated outcome compared with treatment effect using site investigator assessed outcome to give an RTE per trial. These RTEs were pooled in a meta-analysis.
Godolphin^14^	Thesis	Multiple outcomes and outcome types included	Includes Godolphin et al.^2^, Godolphin et al.^19^, Godolphin et al.^22^ and Godolphin et al.^23^
Farrant et al.^21^	Secondary analysis	Composite including stroke (primary outcome) Major hemorrhage (safety outcome)	Compared outcomes determined by both adjudicators and site investigators to determine whether adjudication had any impact on the results of a single trial.
Godolphin et al.^22^	Simulation study	Stoke (primary outcome) Composite including stroke (primary outcome) Functional outcome after stroke (primary outcome)	Simulated systematic error in five trials and random error in simulated trial scenarios. The aim was to identify how much error was required before not adjudicating would change the results of each trial.
Godolphin et al.^23^	Cost–benefit analysis	Stoke (primary outcome) Composite including stroke (primary outcome)	Estimated the cost of adjudication in nine trials. This was compared with the number of outcomes corrected after adjudication.
Van der Ende et al.^24^	Secondary analysis	Functional outcome after stroke (primary outcome)	Compared adjudicated and centrally reported mRS scores to determine the impact of adjudication on the results of a single trial.

mRS: modified Rankin Scale; RTE: ratio of treatment effect.

### Adjudication of stroke or a composite outcome including stroke

We identified four methodological studies that assessed adjudication of stroke or a composite outcome that included stroke (Table 2). One of these, a systematic review and meta-analysis carried out by the present authors,^2^ identified 15 randomized stroke trials in which their primary outcome had been assessed both by site investigators and central adjudicators. For 14 of these trials, the outcome was stroke or a composite including stroke. Similar methodology was followed to the previously described meta-analyses, with the ratio of treatment effects (RTEs) used to quantify the effect of adjudication on trial results. Synthesizing all 14 trials with similar primary outcomes in a fixed-effect inverse variance meta-analysis gave an RTE = 1.02 (95% CI = (0.95–1.10), heterogeneity *p*-value = 1.00).

**Table 2.** Summary of evidence on adjudication in stroke trials.

Study	Trial(s) included	Summary of findings
*Adjudication of stroke or a composite outcome including stroke*		
Ninomiya et al.^16^	PROGRESS	*Stroke* SI HR = 0.74, 95% CI = (0.64–0.85). CA HR = 0.72, 95% CI = (0.62–0.83).
Godolphin et al.^2^	CABACS, ENGAGE AF, ESPRIT, HAEST, ICSS, J-STARS, NASCET, PROGRESS, SOCRATES, SPS3, TARDIS	*Stroke and composite including stroke* Pooled RTE comparing CA and SI = 1.02, 95% CI = (0.95–1.10).
Easton et al.^20^	SOCRATES	*Stroke* SI HR = 0.85, 95% CI = (0.75–0.97). CA HR = 0.86, 95% CI = (0.75–0.97). *Composite including stroke* SI HR = 0.88, 95% CI = (0.78–1.00). CA HR = 0.89, 95% CI = (0.78–1.01).
Farrant et al.^21^	POINT	*Composite including stroke* SI HR = 0.76, 95% CI = (0.60–0.95). CA HR = 0.75, 95% CI = (0.59–0.95). *Ischemic stroke* SI HR = 0.74, 95% CI = (0.58–0.93). CA HR = 0.72, 95% CI = (0.56–0.92). *Major hemorrhage* SI HR = 2.58, 95% CI = (1.19–5.58). CA HR = 2.32, 95% CI = (1.10–4.87).
*Adjudication of functional outcome*		
McArthur et al.^17^	CARS	Agreement between SI and CA at Day 30: weighted *K* = 0.84. Agreement between SI and CA at Day 90: weighted *K* = 0.80.
López-Cancio et al.^18^	REVASCAT	SI cOR = 0.50, 95% CI = (0.30–0.83). CA cOR = 0.57, 95% CI = (0.35–0.95).
Godolphin et al.^2^	REVASCAT	RTE comparing CA and SI = 0.87, 95% CI = (0.43–1.79)
Van der Ende et al.^24^	MR CLEAN	SI cOR = 0.63, 95% CI = (0.45–0.86). CA cOR = 0.60, 95% CI = (0.45–0.83).
*Adjudication of safety outcomes*		
Ninomiya et al.^16^	PROGRESS	*Cause of death (CV vs non-CV vs cancer)* Agreement between SI and CA = 88%, unweighted *K* = 0.79.
Godolphin et al.^19^	ENOS	*SAEs* SI reported patients with SAEs = 1031 (treatment = 522, control = 509). CA reported patients with SAEs = 1022 (treatment = 520, control = 502). ROR for any SAE comparing CA and SI = 0.96, 95% CI = (0.70–1.32). *Likely causality of SAEs* Agreement between SI and CA = 54%, weighted *K* = 0.31.
Easton et al.^20^	SOCRATES	*Cause of death (CV vs non-CV)* Agreement between SI and CA = 92%, unweighted *K* = 0.83. *Bleeding (major vs no major)* Agreement between SI and CA = 88%, unweighted *K* = 0.74.
*Adjudication of baseline covariates*		
Godolphin et al.^3^	ENOS	*Interaction p-values for subgroup analysis by stroke type, observed subgroup effect* Agreement between SI and CA perfect (*K* = 1.00): *p* = 0.39. Agreement between SI and CA good (*K* = 0.78): *p* = 0.40. Agreement between SI and CA poor (*K* = 0.32): *p* = 0.55. *Interaction p-values for subgroup analysis by stroke type, simulated subgroup effect* Agreement between SI and CA perfect (*K* = 1.00): *p* = 0.01. Agreement between SI and CA good (*K* = 0.78): *p* = 0.03. Agreement between SI and CA poor (*K* = 0.32): *p* = 0.16.
*Method of adjudication*		
No stroke-specific evidence identified		
*Blinding status of site assessors*		
Godolphin et al.^22^	HAEST, ICSS, NASCET, REVASCAT, TARDIS	*Blinding is not possible or compromised* Small amount of systematic error needed before trial results change—example, for a binary outcome: between 2.1% and 6% of participants need to be misclassified differentially. *Study is adequately blinded* Large amount of random error needed before trial results change—example, for a trial with binary outcome, 5000 patients, treatment effect (relative risk) = 0.82 and 20% event rate = 64.9% of events need to be misclassified non-differentially.
*Cost of adjudication*		
Godolphin et al.^23^	CABACS, ESPRIT, FASTEST, HAEST, J-STARS, NASCET, PROGRESS, TARDIS, VITATOPS	*Total cost* Range: £2733.18–£135,627.40. *Cost–benefit of adjudication* Mean cost per corrected outcome: £2295.10 (SD =£1482.42).

SI: site investigator; CA: central adjudicator; HR: hazard ratio; cOR: common odds ratio; ROR: ratio of odds ratios; RTE: ratio of treatment effect; CV: cardiovascular; SAEs: serious adverse events; SD: standard deviation.

The remaining three methodological studies were secondary analyses of individual stroke trials (PROGRESS,^4^ SOCRATES,^6^ and POINT^5^). Agreement between adjudicators and investigators for stroke/composite including stroke was excellent for each trial (PROGRESS: stroke = 90%; SOCRATES: stroke = 91%; POINT: composite = 91%). In fact, while there was a small reduction in the number of participants with a primary outcome event in each trial after adjudication, this reduction was similar in both trial arms for all three studies. Furthermore, for all three trials, hazard ratios were almost identical regardless of the outcome assessment (Table 2).

### Adjudication of functional outcome

We identified four methodological studies that reported adjudication of functional outcome in stroke trials. Two studies, our systematic review described previously^2^ and a secondary analysis of an individual trial,^18^ described the same trial REVASCAT.^25^ This trial had a primary endpoint of functional outcome measured on the mRS 90 days post-stroke onset, assessed by both local site investigators and central adjudicators.^18^ Agreement between adjudicators and investigators was 63% (weighted *K* = 0.77) using phone recordings and 87% using video recordings (weighted *K* = 0.92). However, the treatment effect was consistent regardless of assessment (Table 2).

A further study identified was a secondary analysis of the MR CLEAN trial.^24,26^ In this trial, the primary outcome was the mRS at 90 days, and assessments were undertaken via telephone by a research nurse, not explicitly blinded to treatment allocation, with recordings reviewed in a blinded fashion by central adjudicators. Agreement between the research nurse and adjudicators was high (80%), with treatment effects consistent regardless of which mRS result was used (Table 2).

The final study, by McArthur et al.,^17^ suggested that power gains could be achieved by adjudicating functional outcome through increased precision. Increases in power could translate to reduced sample sizes, and this would in turn, lead to cost and time savings. However, through a virtual trial, they assessed agreement between adjudicators and local site investigators on functional outcome and found this excellent, potentially indicating that any power gains through adjudicating may be minimal (Table 2).

### Adjudication of safety outcomes

Many trials adjudicate safety outcomes. For example, ENOS^8^ adjudicated serious adverse events (SAEs),^19^ and in a study by the present authors, we found similar effect estimates using site-assessed or adjudicated SAEs. PROGRESS adjudicated cause of death (Cardiovascular, Cancer or Other Nonvascular) and found global agreement of 88% (unweighted *K* = 0.79) between adjudicators and site investigators.^16^ SOCRATES found overall agreement between adjudicators and site investigators of 88% (unweighted *K* = 0.74) comparing major bleeds and no major bleeding.^20^ However, in ENOS, adjudicators also assessed a more subjective outcome, whether SAEs were related to treatment (Definitely, Probably, Possibly, Unlikely and Definitely not), finding poor agreement of 54% between adjudicators and site investigators^19^ (weighted *K* = 0.31).

### Adjudication of baseline covariates

We identified one methodological study that described the benefit of adjudicating a baseline covariate, in this case stroke type.^3^ This was a study by the present authors, that assessed the ENOS trial and found that results were unchanged regardless of whether stroke type was adjudicated, and only changed when agreement between site investigators and central adjudicators was artificially reduced to extremely low levels (*K* = 0.32, Table 2).

### Method of adjudication

While we did not find any stroke-specific evidence specifically evaluating the method of adjudication, through our assessment of all twelve articles, we identified three main approaches to central adjudication in stroke trials:

1. Adjudicators only assess site reported events.
2. Adjudicators assess site reported events and an additional subset of events.
3. All participants are adjudicated.

A summary of the methods is provided in Supplementary Table 1.

### Trials with blinded outcome assessment versus open-label trials

The 15 trials included in our 2019 systematic review^2^ comprised nine trials where site investigators were blind to treatment allocation and six trials where site investigators were aware of participant’s allocation. There was no indication of an association between blinding status and the RTE. However, the Cochrane review^12^ discussed previously did suggest that there may be an association between the ROR and investigators blinding status (*p* = 0.07). Thus, adjudication may be more worthwhile for studies without adequate blinding.

We identified a simulation study that explored this further.^22^ This study, by the present authors, simulated two distinct scenarios: (1) differential misclassification in the site investigators’ outcome (e.g. an open-label trial) and (2) non-differential misclassification in the site investigators’ outcome (e.g. site-assessment is blinded). In Scenario (1), through simulation based on data from five stroke trials, we found that only a relatively small amount of differential misclassification (range of participants misclassified: 1.9–6%) was needed before adjudicating gives different results to not adjudicating. This simulation demonstrates that trials without sufficient blinding might be at risk of bias unless their outcome is assessed in a blinded fashion (e.g. through central adjudication), although it is important to reiterate that no empirical stroke-specific evidence has ever identified bias that has impacted on eventual trial results and conclusions.

To explore Scenario (2), the study simulated data sets to represent prevention stroke trials with overall event rate ranging from 10% to 50% and number of participants from 1000 to 10,000. The study’s findings were that smaller trials, and those with low event rates were at highest risk of missing a significant treatment effect through not adjudicating, but that for many of the plausible trial settings, the amount of non-differential misclassification needed before a significant treatment effect would be missed was extreme.^22^

### Cost of adjudication

Supplementary Table 2 provides an example of some factors that make up the cost of adjudication. Adjudicating all participants, or identifying additional events will require additional resources, as would employing group adjudication, in which the adjudicators convene together in one location to assess the trial outcome. However, generic adjudication processes are being developed that may help streamline the process.^27^

A retrospective cost–benefit study by the present authors asked trials involved in our previous systematic review^2^ to answer a questionnaire from which they estimated the cost of the adjudication process.^23^ We found that, on average, the adjudication process cost approximately £2300 per decision changed (e.g. stroke to no stroke, or mRS from 3 to 4), with the total cost being more than £100,000 for two of the nine trials.

## Discussion

In this review, including the results of 12 methodological studies evaluating the benefit of central adjudication, we found that adjudication of outcomes in stroke trials rarely has an impact on trial results, but may be important when blinding is compromised or challenging to ensure.

Adjudication may bring benefits on top of ensuring validity of trial findings. It introduces a level of quality control to detect and correct outcomes from poorly trained or poorly performing investigators. Site investigators who require further training could be identified and additional measures provided to help improve their outcome assessment. Adjudication could improve outcome assessment at sites due to a “policing effect,” as site investigators may be aware that their outcome assessment will be checked, and therefore performed more carefully. It has also been reported that the adjudication process brings a level of reassurance to clinical trial staff, with this described as being “of substantial importance.”^16^ However, as shown here, adjudication adds cost and complexity.^28^ With limited resources available for research, it is imperative that trial processes provide sufficient benefit to warrant their cost, so that we avoid research waste.^29^ Before data are sent to adjudicators, a large amount of work is required by trial staff to collect, prepare, and ensure this information does not reveal treatment allocation.^30^ In studies that require multiple adjudications per event, disagreements between adjudicators must be resolved, with a variety of methods currently used to deal with these, each with their own complexities and resource requirements.^31,32^ Furthermore, to date, no stroke trial investigated in this review would have shown differing results if adjudication had not been implemented. Note that for functional outcome, video footage or phone recordings are currently used for adjudication. This has additional complexities for international trials, especially when a common language is not spoken across the trial sites. In this situation, particular dialectic traits may be identified by the local assessors that are not translated through to the adjudicators.

### Suggestions for future stroke trials

We suggest that adjudication may not be necessary for trials with sufficient blinding of outcome assessment, is potentially more beneficial for the most subjective outcomes, and is most important for studies where outcome assessors cannot be blinded. Table 3 summarizes our suggestions for future stroke trials based on their outcome and blinding of site investigators. Our suggestions provided here are general, and we appreciate that the decision to adjudicate may vary substantially based on the trial budget, trial sponsor (e.g. academic/industry), the number of sites, and the experience of the site investigators. However, these suggestions provide a general guide from which to base a decision on whether adjudication is important for a specific trial and outcome.

**Table 3.** Suggestions on when to adjudicate in future stroke trials.

Outcome	SI blind to treatment allocation when assessing outcome	SI not blind to treatment allocation when assessing outcome
Primary outcome: stroke or a composite including stroke	*Adjudication is less important*	*Adjudication is more important*
Primary outcome: functional outcome measured on the mRS	*Adjudication is less important*	*Adjudication is more important*
Subjective key safety or secondary outcome	*Adjudication is less important*	*Adjudication is more important*
Objective key safety or secondary outcome	*Adjudication is not important*	*Adjudication is more important*
Other outcome	*Adjudication is not important*	*Adjudication is not important*

SI: site investigator; mRS: modified Rankin Scale.

### Limitations

This systematic review has several limitations. First, half of the included studies were from the present authors, and it is possible that our research methods and beliefs have unduly influenced the findings. However, the findings from our studies are broadly in agreement from the other articles that we identified. Second, in our previous systematic review,^2^ which provides a large amount of the evidence, we only managed to obtain results from 15 trials. There were a further 74 that were potentially eligible, but did not provide sufficient information to include, even after efforts to contact the authors.^33^ However, without this additional evidence, we have presented the most thorough assessment of adjudication in stroke trials to date. Furthermore, while we did not assess the quality of the randomized trials that were involved in the methodological articles identified in this review, they represent predominantly high-quality prevention stroke trials with commonly used objective outcomes and may not be representative of all stroke trials that adjudicate. Finally, the only assessment of the cost–benefit of adjudication was from a retrospective study.^23^ These costs are only approximate, and a prospective study to properly measure the cost of adjudication is warranted to fully understand the cost and time implications.

## Conclusion

Central adjudication may be of little importance for stroke trials where site investigators are already masked to treatment allocation. However, adjudication may be more important in determining unbiased outcomes for stroke trials without sufficient blinding for site investigators. Given that adjudication comes with non-trivial expense, it is important to have a clear rationale for its inclusion before implementing such a process in future stroke trials. Nevertheless, it is ultimately up to the trialists, funder, and governance teams (sponsor, ethics committee, and regulator) to decide on what degree of adjudication is carried out in any individual trial.

## Supplemental Material

sj-docx-1-wso-10.1177_17474930221094682 – Supplemental material for Should we adjudicate outcomes in stroke trials? A systematic review Supplemental material, sj-docx-1-wso-10.1177_17474930221094682 for Should we adjudicate outcomes in stroke trials? A systematic review by Peter J Godolphin, Philip M Bath and Alan A Montgomery in International Journal of Stroke
