# Supplementary material for: Should we adjudicate outcomes in stroke trials? A systematic review
Source: Int J Stroke. 2022 May 10;18(2):154–62. doi: 10.1177/17474930221094682 (PMC7616431; doi:10.1177/17474930221094682)
Supplement: sj-docx-1-wso-10.1177_17474930221094682 – Supplemental material for Should we adjudicate outcomes in stroke trials? A systematic review [file sj-docx-1-wso-10.1177_17474930221094682.docx]

# Supplementary material

## Full search strategy

Dates searched: 6 November 2018 to 12 October 2021

EMBASE (121 records):

1. exp randomised controlled trial/
2. exp animal/ not human/
3. exp stroke/
4. ((adjudicat* or endpoint or outcome or review or classification or central or event)adj2(adjudicat* or committee or panel or review)).mp.
5. 1 not 2
6. 3 and 4 and 5

MEDLINE (49 records):

1. exp Randomized controlled trial/
2. exp animals/ not exp humans/
3. exp Stroke/
4. ((adjudicat* or endpoint or outcome or review or classification or central or event)adj2(adjudicat* or committee or panel or review)).mp.
5. 1 not 2
6. 3 and 4 and 5

Google Scholar (first 100 records screened):

(“randomised” OR “randomized”) AND "stroke" AND ("adjudication" OR "outcome assessment" OR "event panel" OR "endpoint committee" OR "review committee" OR "central review” OR “event committee”)

**Supplementary Table 1:** Different methods of central adjudication

| **Method** | **Description** | **Pros** | **Cons** |
| --- | --- | --- | --- |
| Adjudication of site-reported events (SR) | Only events identified by the site investigator are sent for adjudication. | - Simple, quick and easy to perform - Least resource intensive - Unbiased if site investigators can always identify Non-events (e.g. never classify a Stroke as No Stroke) | - After adjudication, the number of events can only stay the same or reduce. - If site investigators are poor at identifying events, then these will continue to be missed after adjudication - If site investigators are biased, and not reporting events for specific participants, these biases will remain after adjudication |
| Adjudication of site-reported events and additional events | Events identified by the site investigator are sent for adjudication. In addition, alternative methods (e.g. using computer algorithms to trigger adjudication of events based on prognostic factors) are used to identify additional events. These additional events are also adjudicated. | - Enables adjudicators to identify events that the site investigator missed - Bias is always less than or equal to SR method | - Method is similar to SR if methods to identify additional events are not effective - Still open to bias if site investigators are poor at identifying Non-events and methods to identify additional events are poor - Additional methods may require lots of additional adjudication and resource |
| Adjudication of all participants | All participants are adjudicated at some designated time-point | - Allows a completely blinded assessment of every participant - Least bias of all three methods | - Very challenging and resource intensive for large trials - Difficult for trials in which the outcome is not measured at a fixed time-point (e.g. most prevention trials) |

**Supplementary Table 2:** Example of the processes required to facilitate central adjudication of site-reported events, with disagreements between the site investigator and central adjudicator settled by an additional adjudicator

| **Process** | **Frequency** | **Resource used** |
| --- | --- | --- |
| Website creation | Once, often at the start of the trial | Co-ordinating centre (database programmer to develop website) |
| Obtain trial data for adjudication | Per participant/event | Co-ordinating centre (trial staff to manage obtaining the information) and Trial sites (research staff to send the original source data) |
| Anonymising source material | Per participant/event | Co-ordinating centre (trial staff to check data) |
| Adjudicating event/outcome | Per adjudication | Adjudicator (time) and Co-ordinating centre (direct payment e.g., £20) |
| Dealing with queries from adjudicators | Reliant on the quality of the original source data. | Co-ordinating centre (trial staff to correspond with queries) |
| Obtaining additional information requested by adjudicators | Reliant on the quality of the original source data. | Co-ordinating centre (trial staff to request and manage obtaining the data) and Trial sites (research staff to retrieve and send the additional information) |
| Disagreement between adjudicators and site investigator | Reliant on the outcome, experience of the site investigators and quality of the data, but disagreements occur in approximately 10% of cases | Second adjudicator (time) and co-ordinating centre (direct payment, trial staff to identify disagreement and arrange and manage second adjudication) |
| Administration following adjudication (e.g. filing documents, outcome of adjudication and decisions made regarding disagreements) | At the end of the adjudication process | Co-ordinating centre (trial staff to physically file all relevant information) |
